# A long-term complication of clitoral cyst after female genital mutilation

**DOI:** 10.11604/pamj.2023.46.23.31939

**Published:** 2023-09-14

**Authors:** Özgür Şahin, Erol Nadi Varlı, Abdirahman Omar Moallim, Harun Egemen Tolunay

**Affiliations:** 1 Recep Tayyip Erdogan Somalia Mogadishu Training and Research Hospital, Department of Obstetrics and Gynecology, Mogadishu, Somalia,; 2 Etlik Zübeyde Hanım Maternity and Women´s Health Teaching and Research Hospital, Perinatology Department, Ankara, Turkey

**Keywords:** Female genital mutilation, long-term complications, clitoral cyst, awareness of female genital mutilation

## Abstract

Female genital mutilation (FGM) was seen in 30 countries, especially in Africa and also in Asia and the Middle East. According to WHO data, Somalia is where FGM is performed most frequently. Our study aimed to evaluate the recordings of patients with FGM who were diagnosed with a traumatic clitoral cyst. We identified the clitoral cyst cases between February 2015 and August 2020. We collected clinical, surgical, sociodemographic, and histopathological details such as age, marital status, patient resume, age at which FGM was performed, complaints, size of the cyst consultation reasons, FGM procedural long-term complications, sexual function, husband polygamic relationship status, and histological findings. A total of 21 patients diagnosed with clitoral cysts were included in the study. The technique was easily applied in every patient, and the cysts were removed intact, except in 2 patients. There were no intraoperative complications; only minimal bleeding was seen. Except for one patient, all had unilocular cysts, and the final pathological examination revealed an epidermal inclusion cyst. We observed a neuroma developed due to genital trauma due to FGM in one of our patients. Female circumcision and its consequences are not familiar to many healthcare professionals in the developed world. We want to increase awareness of female circumcision and its long-term complication of clitoral cysts among healthcare professionals worldwide.

## Introduction

Traditional female genital mutilation (FGM), previously known as female genital mutilation, has received increasing international attention in recent years. Since the 1990s, NGOs (Non-Governmental Organizations) such as the World Health Organization (WHO) [1], the United Nations [2] and UNICEF (United Nations International Children's Emergency Fund), the European Union [3] and the United Kingdom Women's Health Research and Development Foundation (FORWARD UK) [4] have been instrumental in establishing a strong global agreement against the continuity of this practice. There are approximately 200 million women who have FGM procedures around the world (Table 1). Approximately 3 million procedures are performed on baby girls and young girls each year, especially in African countries [5]. Somalia is the country where female genital mutilation is performed most frequently, according to WHO data. The type of procedure performed varies mainly depending on ethnic origin. The most common type of procedure is Type I (clitoridectomy), which was 90% of all procedures with Type II (excision) and IV (“nicking” without flesh removed). More than 8 million women undergo infibulation (Type III) (Table 2). Infibulation, the most severe form of female genital mutilation, is especially seen in the north of Africa: Somalia, Sudan, Djibouti, and Ethiopia [6]. Understandably, solving this problem has proven to be not as easy as expected due to the complex nature and impact on a wider audience in society. Currently, the implementation appears to be predominantly African-centered. More than 30 countries are affected. Somalia is also among these countries [7]. Clitoral cyst development, especially after type II - III FGM, is one of the most common long-term complications [8]. Generally, cyst formation is caused by the burial of keratinized epithelial cells and sebaceous glands in the scar line, but neuromas may also develop due to genital trauma resulting from FGM [9,10]. The Objectives of this study, were to evaluate the treatment of this pathology in light of current guidelines by reviewing the clitoral cyst cases after FGM that we encountered in our hospital and draw attention to this issue again.

**Table 1:** prevalence of female genital mutilation in nineteen countries (adapted from WHO data, 2016)

Country	Prevalence
Benin	13%
Egypt	91%
Ethiopia	74%
Kenya	27%
Mali	89%
Nigeria	27%
Sudan	88%
Tanzania	15%
Iraq	8%
Yemen	23%
**Somalia**	**98%**
Senegal	26%
Mauritania	69%
Guinea	96%
Sierra Leone	88%
Cote d'Ivoire	38%
Liberia	66%
Burkina Faso	76%
Gambia	76%
Total of 19 Countries	**Total Number: 200 million Girls and Women**

**Table 2:** female genital mutilation types-their explanations (adapted from WHO data, 2020-February)

FGM Type	Explanation
**Type I**	Total or partial removal of the clitoris and/or the prepuce (clitoridectomy)
**Type II**	Total or partial removal of the clitoris and/or prepuce with partial or total excision of the labia minora and majora
**Type III (infibulation)**	Involves excision of part or all of the labia minora and/ or majora, and stitching/narrowing of the vaginal opening, with or without removal of the clitoris (pharaonic circumcision)
**Type IV**	Includes all other procedures, also cauterization by burning of the clitoris and surrounding tissues

## Methods

**Study type and design:** between February 2015 and August 2020, we conducted a retrospective, descriptive study to evaluate all the clitoral cyst cases after FGM encountered in the Department of Gynecology of the Recep Tayyip Erdogan Somalia Mogadishu Training and Research Hospital, Mogadishu, Somalia.

**Setting:** after the approval was obtained from our local committee, the files of the patients hospitalized in our Gynecology department with clitoral cysts diagnosis between 2015 and 2020 were evaluated retrospectively. Since 2015, our clinic has seen around 2000 women per month for various reasons, including surgical treatments such as defibrillation and clitoral reconstruction. In the literature, according to the Foldès technique, clitoral reconstruction has been used since January 2013 [11]. Foldès' practice includes resecting the FGM cutaneous scar covering the clitoral stump; dissection of the clitoris up to the elbow, and removing subcutaneous periclitoral fibrosis [10].

**Methods of collecting data and instrumentation:** in our hospital, follow-up examinations and multidisciplinary care, including sexual health education as well as clitoral reconstruction and FGM, are performed for women who request this procedure. We evaluated all patients who attended our clinic. Electronic health records of patients were used to collect data. We collected sociodemographic, surgical, clinical, and histopathological data. The information included marital status, age, patient resume, age at which FGM was performed, complaints, size of the cyst, reasons for consulting our clinic, long-term complications of FGM (e.g., vulvar pain or dyspareunia), sexual function, husband polygamic relationship status, clinical findings (e.g., FGM/ type), and histological findings.

**Method of data analysis:** before the data analyses, all data were checked to detect anomalies and inaccuracies. Continuous variables are reported as means with standard deviation (SD), maximum, minimum, and median values. Categorical variables are reported as percentage and frequency. After the data were entered in the EXCEL file, they were transferred to IBM SPSS.23 program and evaluated by statistical analyses.

**Availability of data and materials:** if the manuscript is approved, the corresponding author will, upon reasonable request, make the datasets used during the inquiry available.

**Ethics approval and consent to participate:** the Local Ethics Committee of the Recep Tayyip Erdogan Somalia Mogadishu Training and Research Hospital, Mogadishu, Somalia, granted its approval for the study's conduct, protocol, and procedures (approval number: 02-20-221). All participants read the purpose statement of the study. A written and informed consent form was received from the patients.

## Results

**Figure 1:**
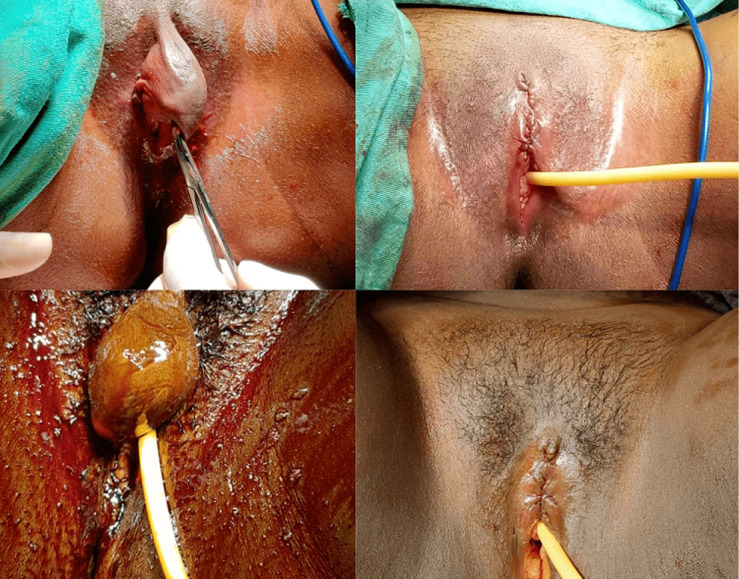
preoperative-postoperative photos of cases

The study included a total of 21 patients diagnosed with a clitoral cyst. The technique was easily applied in every patient, and the cysts were removed intact, except in 2 patients, without minimal bleeding and intraoperative complications. Except for one patient, all patients had unilocular cysts. And the last pathological examination revealed an epidermal inclusion cyst. In one of our patients, we observed that a neuroma developed due to genital trauma due to FGM. All patients were discharged on the second postoperative day. In the follow-up of the patients, it was observed that the symptoms did not recur (Figure 1).

**Findings:** the mean age of the patients was 33.00±14.89. Mean values for BMI, gravida, parity, live births, and abortion were as follows; 27.05±2. 55, 3.24±4.23, 3.19±4.18, 3.14±4.21, and .05±.22, respectively. The mean size was found as 5.62±1.77. While the average FGM date (age) was found to be 7.33±1.77 years old, the average start date of cyst growth (age) was found as 18.05±8.18 years old (Table 3). The female genital mutilation type of 18 (85.7%) of the patients was type II. However, all patients were diagnosed with a clitoral cyst. The most common type of complaint was discomfort mass in the vagina. The pathology type of most patients was an epidermal cyst. 66.7% of female genital mutilation type II patients were married (Table 4).

**Table 3:** median, minimum, maximum, and mean, standard deviation values of some medical and sociodemographic parameters of patients

Parameters	Number	Mean	Standard Deviation	Minimum	Maximum
Age	21	33.00	14.89	19	73
BMI	21	27.05	2.55	22.5	31.8
Gravida	21	3.24	4.23	0	11
Parity	21	3.19	4.18	0	11
Number of Livebirths	21	3.14	4.21	0	11
Abortion	21	.05	.22	0	1
Size (cm)	21	5.62	1.77	4	10
FGM date (age)	21	7.33	1.77	5	12
Start date of cyst growth (age)	21	18.05	8.18	8	48

**Table 4:** frequency and percentage of some medical and sociodemographic parameters of patients

Female Genital Mutilation Types of Patients	Female Genital Mutilation Type 2 (18 patients)	Female Genital MutilationType 3 (3 patients)
**Complaints**		
Asymptomatic	7(%38.8)	0
Discomfort mass in the vagina	11(%61.2)	2(%66.7)
Heaviness at vulva	0	1(%33.3)
**Pathology**		
Epidermal inclusion cyst	15(%83.3)	3(%100)
Neuroma	1(%5.6)	0
Ruptured epidermal cyst	2(%11.1)	0
**Marital status**		
Single	6(%33.3)	3(%100)
Married	12(%66.7)	0
**Husband polygamic status**		
No	10(%55.6)	1(%33.3)
None(Single)	6(%33.3)	0
Yes	2(%11.1)	2(%66.7)

## Discussion

Several short and long-term complications of FGM have been described from different types in the literature. The frequency of these complications is related to the type of FGM and the specialist experience. Higher complication rates were seen, especially in more severe forms. The most common is forming an epidermal inclusion cyst in the long term after type III infibulation. In 1992, Dirie *et al*. reported that 36 (12.4%) women among 290 women in Mogadishu, the capital of Somalia, had post-infibulation epidermal inclusion cysts [12]. Female circumcision is usually performed by people specially appointed for this job, without anesthesia and in bad conditions. This can lead to additional undesirable damage after female circumcision, with complications developing later.

Our study revealed the long-term sequelae formation after type 2-3 FGM. Patients who have undergone FGM have dyspareunia more than women who have not been exposed to FGM [13]. Female sexual dysfunction is severely associated with sexual desire, inability to achieve orgasm, impaired arousal, sexual pain disorder, or a combination of these issues resulting in other significant psychosomatic distress. Sexual dysfunction after female circumcision is a crucial issue. Women with female genital mutilation experience a wide variety of health problems, including poor quality of sexual life, which can even cause marital problems [14]. Prolonged symptoms and the difficulty of these girls and women to access medical assistance delay full recovery after treatment. Therefore, increased awareness of long-term complications after female genital mutilation is required.

## Conclusion

FGM was seen in 30 countries, especially in Africa and also in Asia and the Middle East. Some forms of FGM were also seen in other countries. Moreover, with the spread of immigration, the number of girls and women living outside their countries of origin who have had female genital mutilation or are at risk in Europe, Australia, and North America has increased. Despite this, FGM and its consequences are not familiar to many healthcare professionals in the developed world. With this point of view, we want to increase the awareness of female genital mutilation and its long-term complication of clitoral cysts among healthcare professionals worldwide and harm as few women as possible from this traumatic situation, whose long-term complications we share in our case series.

### What is known about this topic

- *Female genital mutilation (FGM) is seen in 30 countries, mainly in Africa;*
- *Various short-term and long-term complications of FGM that we investigated in our study have been described in the literature*.

### What this study adds

- *Somalia is the country where female genital mutilation is most common;*
- *With increasing immigration, we aim to raise awareness among healthcare professionals worldwide about female genital mutilation and its long-term complication, a clitoral cyst*.
